# Enhancing the Performance of Hemihydrate Phosphogypsum by the Collaborative Effects of Calcium Hydroxide and Carbonation

**DOI:** 10.3390/ma17102204

**Published:** 2024-05-08

**Authors:** Jiawen Huang, Zanqun Liu, Xiangsong Wei, Xiaojiang Ding, Jiahui Zhu, Yilin Zhao, Babar Iqbal, Shulai Guo

**Affiliations:** 1 School of Civil Engineering, Railway Campus, Central South University, No. 68, South Shaoshan Road, Tianxin District, Changsha 410018, China; 214811013@csu.edu.cn (J.H.); zhujiahui0108@csu.edu.cn (J.Z.); 214811032@csu.edu.cn (Y.Z.); babarkhan04@csu.edu.cn (B.I.); gsl2023@tongji.edu.cn (S.G.); 2 Geology Institute of China Chemical Geology and Mine Bureau, Block B, No. 19, Xiaoying Road, Chaoyang District, Beijing 100101, China; diyanyuan123@126.com

**Keywords:** hemihydrate phosphogypsum, calcium hydroxide, carbonation curing, water resistance, compressive strength, carbon footprint

## Abstract

Normally, the acidic impurities in hemihydrate phosphogypsum (HPG) must be neutralized when HPG is utilized, and a little amount of calcium hydroxide (CH) is the best choice. In this paper, the effects of excessive CH (5 wt.%, 10 wt.%, 15 wt.% and 20 wt.% of HPG) for carbonation curing on the performance of hardened HPG paste were studied. According to the results of macro tests and microanalyses of XRD, TG, SEM-EDS, MIP and N_2_ physisorption, it could be verified that CaF_2_, Ca_3_(PO_4_)_2_ and a large amount of nanoscale CaCO_3_ crystals were produced as a result of neutralization and carbonation, and the compressive strength and the water resistance of carbonated HPG + CH paste were significantly improved due to the effects of nanoscale CaCO_3_ crystals on pore refinement and the coverage on the surfaces of gypsum crystals of the hardened paste. Therefore, this study suggests a feasible and green method for recycling HPG/PG, with the collaborative effects of neutralization, performance enhancement and reductions in CO_2_ emissions.

## 1. Introduction

Phosphogypsum (PG) is an industrial solid waste obtained in the process of phosphoric acid production [1], and its main component is calcium sulfate dihydrate (CaSO_4_·2H_2_O), the same as natural gypsum, and PG represents a renewable gypsum resource [2]. However, PG contains amounts of fluorine, phosphorus, organic matter and other impurities [3] that hinder the utilization of PG [4]. According to statistics, the global PG production is around 280 million tons per year [5], while the comprehensive utilization rate is only 15% [6]. The remaining PG is accumulated [7], not only taking up lots of land [8], but also causing serious environmental issues [9]. Therefore, the effective disposal and reuse of PG offer benefits for the environment, economy and society.

When applied to building materials, PG can be used as a cement retarder [10], a component of composite binding material [11] and a gypsum building material [12]. The consumption of PG in cement retarders and composite binding materials is limited. Calcinating PG to produce hemihydrate phosphogypsum (HPG) to prepare gypsum building material is one of the most effective ways to utilize PG, and the main component of HPG is calcium sulfate hemihydrate (CaSO_4_·0.5H_2_O), which can be made into gypsum board, gypsum block and other decorative or functional building products [13]. However, the impurities of phosphorus and fluorine in PG affect the crystallization process of gypsum during the hydration of HPG [14], resulting in poor strength and a loose microstructure of hardening pastes [15], and HPG products generally have poor water resistance owing to the high solubility of CaSO_4_·2H_2_O and the loss of strength [16].

To reduce the negative effects of impurities, alkaline regulators are generally used, such as calcium hydroxide (Ca(OH)_2_, (CH)), magnesium hydroxide (Mg(OH)_2_), ammonium hydroxide(NH_3_·H_2_O), sodium hydroxide (NaOH), cement [17], and so on. All alkaline regulators could neutralize acidity, but the effects of Mg(OH)_2_, NH_3_·H_2_O and NaOH on HPG strength are not obvious, and cement could reduce the volume stability of HPG [17]. Adding CH is the best choice to modify HPG, because 0.1–2.0 wt.% CH has been shown to contribute to the immobilization of impurities [18], promote hydration [17], regulate the growth of gypsum crystal [19], optimize performance [20] and elevate the potential for carbon absorption. CH (or industrial solid wastes with CH as the main content, such as calcium carbide slag) carbonation, as an important CO_2_ sequestration technology, can generate insoluble calcium carbonate (CaCO_3_) crystals [21].

The carbonation of cement and concrete has been studied extensively. The early-age carbonation of cement can form carbonate binders composed primarily of CaCO_3_ and amorphous silica/alumina gel on the surface, which improve pore structure, decrease permeability and achieve lower carbon emissions [22]. PG could be carbonated to prepare CaCO_3_ in the presence of ammonium ions [23], but it struggles to be carbonated under normal circumstances. By adding excessive CH into HPG and combining with carbonation curing, after the neutralization reaction, the remaining CH could be carbonated and form a lot of insoluble CaCO_3_, which may fill the pores of the hardened HPG paste to further improve its performance, similar to the early carbonation of cement. So, the novelty of this work is in combining the needs of HPG materials regarding impurity solidification, performance improvement and carbon emission reduction.

Therefore, CH, carbonation curing and their collaborative effects on the performance of HPG, including setting and hardening properties, hydration products, microstructure development, carbon footprint and energy consumption, were evaluated in this study. The hydration products of the hardened pastes were measured by X-ray diffraction (XRD) and thermogravimetric analysis (TG). A scanning electron microscope (SEM) was used to observe the microstructure. N_2_ physisorption and mercury intrusion porosimetry (MIP) were both used to measure the pore structure. These conclusions were suggested to provide guidance for the comprehensive utilization of PG and CH-containing solid wastes.

## 2. Materials and Experiments

### 2.1. Raw Materials

The hemihydrate phosphogypsum (HPG) used in this study was donated from Guizhou Phosphating Green Environmental Protection Co., Ltd. (Guiyang, China) and is produced by crushing, flotation, washing, pre-drying by hammer air dryer, calcining by three boiling calciners connected in series, cooling, grinding modification, homogenization, storage and other processes. The physical and mechanical properties of HPG are shown in Table 1. The internal exposure index I_Ra_ and the external exposure index I_c_ evaluated the radioactivity level of HPG. It can be found that the radioactivity is lower than the criteria of the Chinese standard (GB 9776-2022) [24], which means HPG is safe for use as a construction material [25].

**Figure 1:**
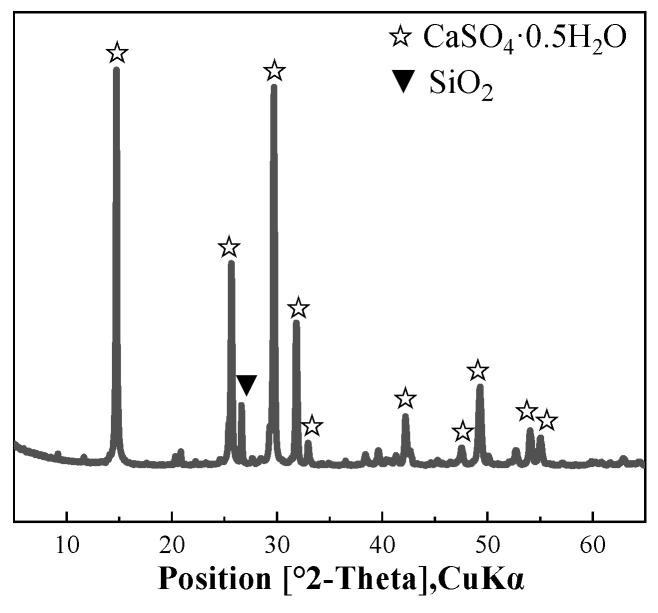
XRD pattern of HPG.

The mineral compositions of HPG are shown in Figure 1 according to XRD analysis, and the primary crystalline phases in HPG are hemihydrate gypsum (CaSO_4_·0.5 H_2_O) and quartz (SiO_2_).

The chemical compositions of HPG powder as characterized by XRF analysis are shown in Table 2.

**Figure 2:**
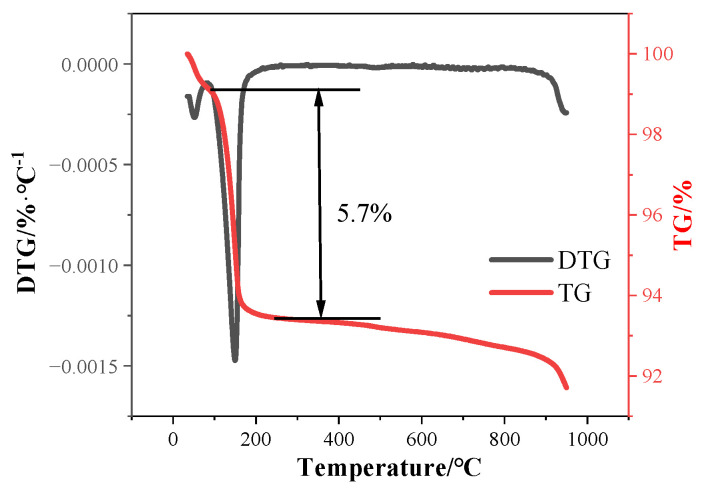
TG/DTG curves of HPG.

The main chemical compositions are CaO and SO_3_, which are the specific components of CaSO_4_·0.5H_2_O. There are also two specific impurities, F and P. The chemically combined water in HPG is 5.7%, as suggested by the TG-DTG analysis shown in Figure 2, and the content of hemihydrate gypsum (CaSO_4_·0.5H_2_O) is 91.8%.

**Figure 3:**
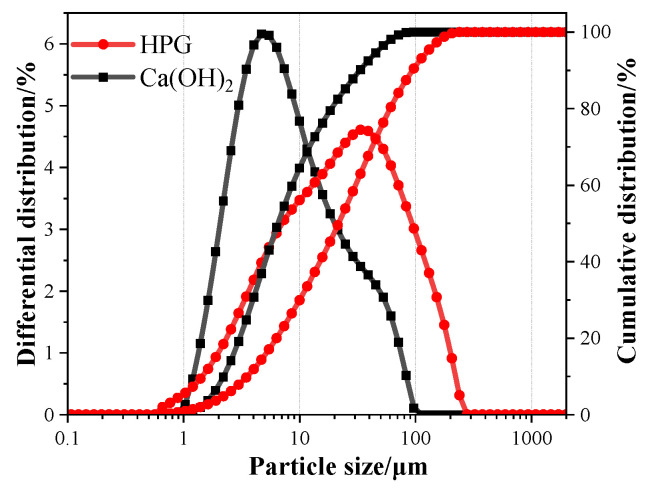
Differential and cumulative particle size distribution curves of the raw material (tested during the dispersion of ethanol).

The particle size distributions of HPG and calcium hydroxide (CH) were determined by the laser particle size analyzer shown in Figure 3. The particle size distribution range of HPG is relatively wide, with almost 80% distributed between 1 and 70 μm; there is a median particle size of 21.7 μm and the specific surface area is 214.14 m^2^/kg.

**Figure 4:**
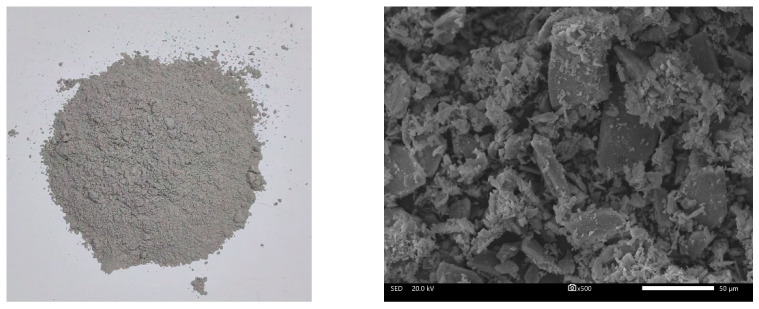
Particle morphology and SEM image of HPG.

The particle morphology and SEM image of HPG are presented in Figure 4, showing that the powder is grey and HPG is composed of CaSO_4_·0.5H_2_O crystals with irregular shapes and uneven boundaries.

For the exact quantitative analysis, analytically pure-grade calcium hydroxide (CH) was used in this work, composed of more than 95% Ca (OH)_2_ and obtained from Tianjin Zhiyuan Chemical Reagent Co. Ltd. (Tianjin, China) The chemical compositions of CH are illustrated in Table 2. As shown in Figure 3, the particle size of CH is finer than that of HPG, with 6.5 μm median particle size and 1222.84 m^2^/kg specific surface area. Retarder XK (a modification of amino acid retarder-200p) obtained from Swiss Sika Group was used to improve the working ability of HPG.

### 2.2. Sample Preparation and Curing

The mix proportions of HPG and CH are presented in Table 3, designated as CH-0, CH-5, CH-10, CH-15, and CH-20, containing 0 wt.%, 5 wt.%, 10 wt.%, 15 wt.%, and 20 wt.% CH as the HPG replacement, respectively.

The water-to-binder ratios of the pastes are all 0.58, which is the water requirement for the normal consistency of HPG. The water was deionized water and was stored at 20 ± 2 °C for 24 h. The addition of 0.01% retarder XK of the mass of HPG was used to increase the setting time and ensure the casting process.

For each mix, 40 mm × 40 mm × 160 mm paste prisms were cast in accordance with GB 17669.3-1999 [26]: XK and Ca(OH)_2_ were mixed in water for 2 min to obtain a homogeneous suspension liquid. Then, HPG was added to the liquid with 5 min of mixing, and the prepared paste was poured into the molds. Subsequently, the molds were repeatedly vibrated until no air bubbles were produced. The specimens were cured at 20 ± 2 °C, and 70 ± 5% relative humidity for 1 h, and then demolded. Once demolded, one set of paste prisms was exposed to a carbonation environment (20 ± 2% CO_2_ concentration, 70 ± 5% R.H, 20 ± 2 °C) for 14 days. For comparison, the second set of paste prisms were cured in atmosphere (approximately 0.04% CO_2_ concentration, 70 ± 5% R.H, 20 ± 2 °C) for 14 days.

### 2.3. Test Procedures

#### 2.3.1. Water Requirement for the Normal Consistency, Fluidity and Setting Time

Then, 300 g of powder and water were stirred for 30 s. Then, the slurry was poured into a steel cylinder with a 50 mm diameter and 100 mm height, and the cylinder was lifted quickly. Subsequently, the diameters of the slurry in the two vertical directions were measured and the average value was taken as the fluidity. The water requirement for normal consistency was confirmed when the fluidity was equal to 180 ± 5 mm. In this paper, the water requirement for the normal consistency of HPG was 0.58.

The initial setting time and final setting time of the pastes were determined using the Vicat apparatus. The point at which the needle could no longer touch the bottom of the glass plate was taken as the initial setting time of the pastes. When the depth of the needle inserted into pastes was no more than l mm, this was taken as the final setting time of the pastes.

According to Chinese standard GB/T 17669.4-1999 [27], the normal consistency water requirement data used and the setting time data used are the mean values of at least two test results.

#### 2.3.2. Mechanical Properties

The mechanical properties were tested according to GB/T 17669.3-1999 [26] in China. The specimens were dried at 40 ± 2 °C until they reached a constant weight when the mass difference between them at 24 h was less than 0.2%. The compressive strength of the samples was measured at a 0.6 kN/s loading speed on a TYA-300B testing machine. The compressive strength data used are the mean value of at least five test results after excluding the results with deviation over 15%.

#### 2.3.3. Water Resistance

The strength of HPG pastes under saturated conditions, the softening coefficient and the saturated water absorption rate were used to evaluate water resistance. The drying samples were soaked in water (20 ± 2 °C) for 1 day. A damp towel was wrung out to remove excess water, and we then wiped away the water on the surfaces of samples. The weight and the strength of saturated specimens were tested immediately. The softening coefficient can be calculated using Equation (1) and the saturated water absorption rate can be calculated by Equation (2). Three samples were used in the test.

(1) $$S=\frac{S_{W}}{S_{D}}$$

(2) $$R=\frac{m_{W}-m_{D}}{m_{D}}\times 100\%$$

where *S* is the softening coefficient; *S_W_* (MPa) is the compressive strength of specimens under saturated conditions; *S_D_* (MPa) is the compressive strength of drying samples; *R* is the water absorption at saturation; *m_W_* (g) is the quality of specimens under saturated conditions; and *m_D_* (g) is the quality of drying specimens.

#### 2.3.4. Carbonation Area

After curing for 14 days, the hardened HPG pastes were split in the middle and applied 1 wt.% phenolphthalein pH indicator ethanol solution on the fresh split surface, to examine the carbonation area.

#### 2.3.5. Chemical Composition Analysis

The raw materials were dried to constant weight in a vacuum at 40 °C and were ground with a mortar and pestle. The chemical composition analysis of raw materials was conducted on an X-ray fluorescence spectrometer (Bruker, S8 TIGER, Billerica, MA, USA).

#### 2.3.6. Particle Size Distribution Analysis

The raw materials were dried to a constant weight in a vacuum at 40 °C. The particle size distribution analysis of raw materials was performed using a laser particle size analyzer (OMEC, Topsizer, Zhuhai, China) and the dispersion medium was ethanol.

#### 2.3.7. Hydration Products Analysis

After curing for 14 days, the hardened pastes were crushed into pieces of less than 5 mm. In order to terminate hydration, the pastes were immersed in isopropanol for 7 days and then dried in a vacuum for another 7 days. The drying pastes were ground with a mortar and pestle to prepare powder samples.

The crystalline phases in the powder samples were measured using an X-ray diffraction machine (Rigaku, model D/max·III·X, Tokyo, Japan) with Cu Kα radiation (λ = 1.5418 Å) at 40 KV and 40 mA, a scanning range of 5–65°, a scanning step of 0.02° and a scanning rate of 2°/min. Table 4 presents the phases used with a PDF code and an ICSD collection code.

The powder samples were characterized by TG-DTG using a Germany Netsch STA 449C thermal analyzer with a temperature range of 35–950 °C. The heating rate was 10 °C/min and the nitrogen gas flow was 20 mL/min. Water lost before and after 100 °C is considered as adsorbed water and chemical combined water, respectively.

#### 2.3.8. Microstructure Analysis

A JSM-IT500L machine (JEOL, Tokyo, Japan) was used for the scanning electron microscopy (SEM) test to analyze the microstructures of the pastes after 14 days of curing. The drying fractured samples were platinum sputter-coated. Images were acquired at 20 kV and 40 μA. EDS analyses were performed at 20 kV and 66 μA, and image magnification ranged from 50 to 20,000 times.

#### 2.3.9. Pore Structure Analysis

After curing for 14 days, the hardened pastes were drilled into cores to make the samples for pore structure analysis. Hydration also had to be terminated in the samples. Mercury intrusion porosimetry (MIP) was conducted on a AutoPoreIV9500 apparatus (Micromeritics, Norcross, GA, USA). N_2_ physisorption measurement was performed using a Builder SSA-4000 apparatus (Builder, Beijing, China) and analyzed using a BJH model [34].

## 3. Results

### 3.1. Effects of CH Addition on the Setting Time and Fluidity of HPG Pastes

**Figure 5:**
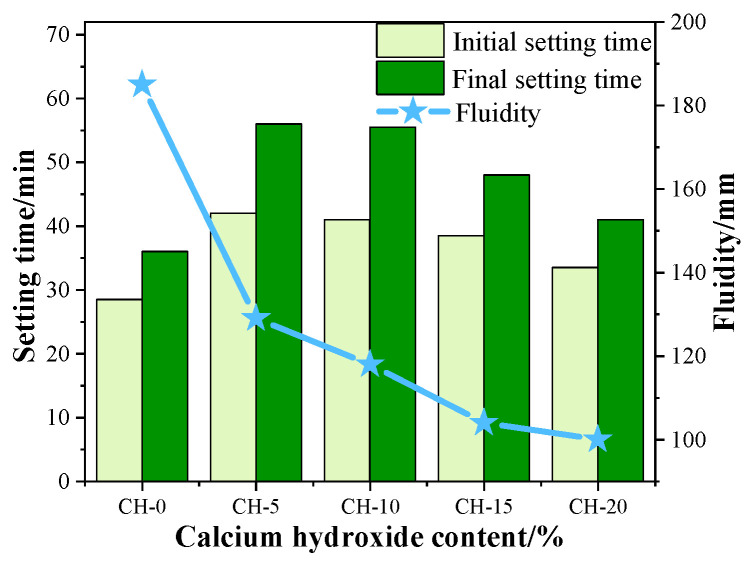
Effect of CH addition on the setting time and the fluidity of HPG paste.

Figure 5 shows the setting properties of HPG pastes with variable additions of CH. It can be seen that CH additions prolonged the setting time of HPG pastes, and the setting time presented a tendency of increasing first and then decreasing with the increase in CH. The HPG paste with 5 wt.% CH showed the longest setting time, with 42 min of initial setting time and 56 min of final setting time. The group without CH had the shortest setting time, with a 28.5 min initial setting time and 36 min final setting time. As to the fluidity, when the CH increased from 0 wt.% to 20 wt.%, the fluidity reduced from 185 mm to 100 mm. The main reason for the reduced fluidity is that CH has a finer particle size and a larger surface area than HPG, and it requires more water to achieve the same consistency.

### 3.2. Effects of Carbonation on the Mechanical Properties and Water Resistance of HPG + CH Pastes 

**Figure 6:**
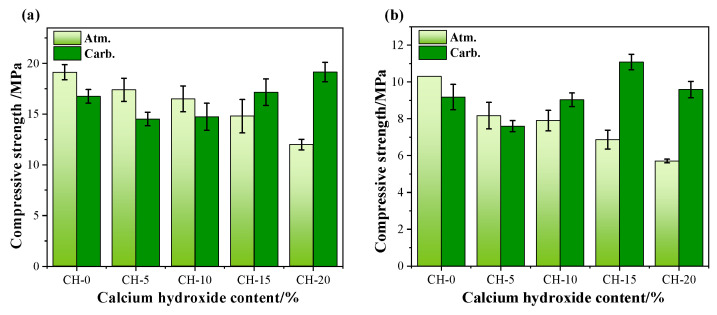
Effects of CH addition and carbonation on the compressive strength of HPG paste. (**a**) dry conditions, (**b**) saturated conditions.

Figure 6 displays the effects of CH addition and carbonation curing on the compressive strength of HPG pastes. 

From Figure 6a, it can be seen that the compressive strength of specimens under atmosphere showed a downward trend with the increase in CH addition by approximately 9–37%, which suggests that excessive amounts of CH could reduce the strength. However, the compressive strengths of samples under carbonation curing first decreased and then increased with the increase in CH. The compressive strengths of CH-15 and CH-20 under carbonation curing were 17.16 MPa and 19.15 MPa, respectively, which increased by 16.2% and 59.6% compared to those pastes under atmosphere. After 1 day of water immersion, as shown in Figure 6b, the compressive strength of the pastes showed a similar trend to the dry condition, whereby the CH addition decreased the compressive strength, but the carbonation curing was shown to significantly increase the compressive strength of HPG + CH pastes, especially the carbonation-cured CH-15, which showed a 60.9% increase compared to the CH-15 under atmosphere curing.

**Figure 7:**
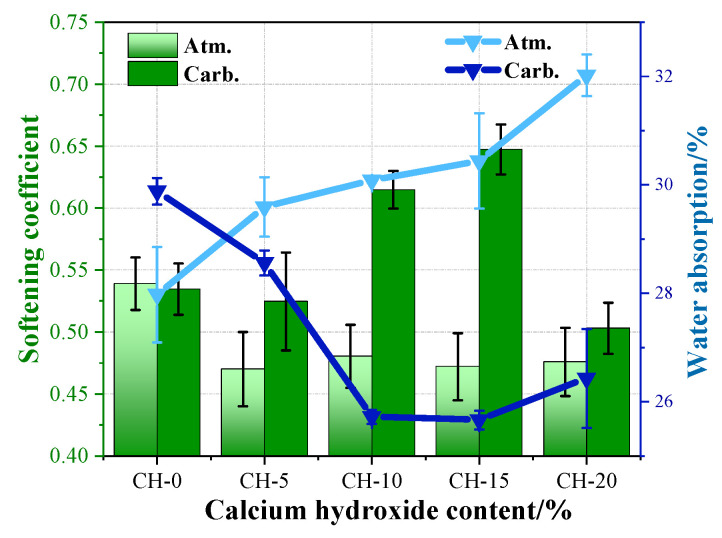
Effects of CH addition and carbonation on the softening coefficient and the water absorption rate of HPG pastes.

The high-water resistance of gypsum products will encourage their use. The softening coefficient and the water absorption rate were calculated and illustrated in Figure 7.

It can be seen in Figure 7 that: (1) The CH addition could decrease the water resistance of HPG pastes with a small softening coefficient and a high water-absorption rate. (2) The carbonation curing also decreased the water resistance of pure HPG paste, but could markedly improve the water resistance of HPG + CH pastes, especially for the softening coefficient of carbonation-cured CH-15, which reached 0.65. (3) The inadequate water resistance of CH-20 may be due to too much CH addition, and the CaCO_3_ formation under carbonation curing could not make up the loss of water resistance of HPG paste. Pore structure analysis helped us to identify the causes.

### 3.3. Carbonation Area of HPG + CH Pastes

**Figure 8:**
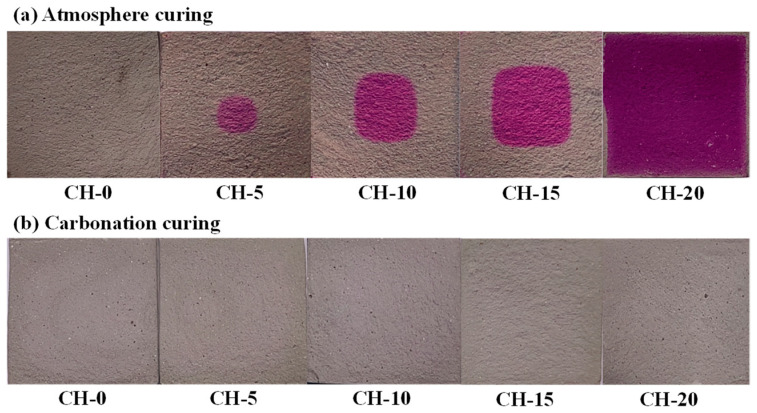
Fresh split surfaces of specimens sprayed with a 1 wt.% phenolphthalein indicator ethanol solution: (**a**) atmosphere-cured specimens; (**b**) carbonation-cured specimens.

Figure 8 illustrates the freshly split surfaces of specimens sprayed with a 1 wt.% phenolphthalein indicator ethanol solution after atmosphere curing and carbonation curing for 14 d.

In Figure 8a, the red color shown in the center of atmosphere-cured specimens containing CH indicates that carbonation proceeds from the outsides to the insides. Under atmosphere curing, the CH in the periphery of specimens was carbonated due to natural carbonation, and uncarbonated CH occurred in the centers of the specimens. Increasing the CH addition enlarged the red areas, which means that the carbonation rate slowed down with the increase in CH addition. In Figure 8b, for all specimens with CH exposed to the carbonation environment, no obvious color difference appeared on the split surfaces. This reflects that CH in the specimens has been neutralized by CO_2_ after carbonation curing for 14 d. XRD and TG-DTG analysis helped us to further identify the reaction products in the pastes.

### 3.4. Products Identification of HPG + CH Pastes

#### 3.4.1. XRD Analysis

**Figure 9:**
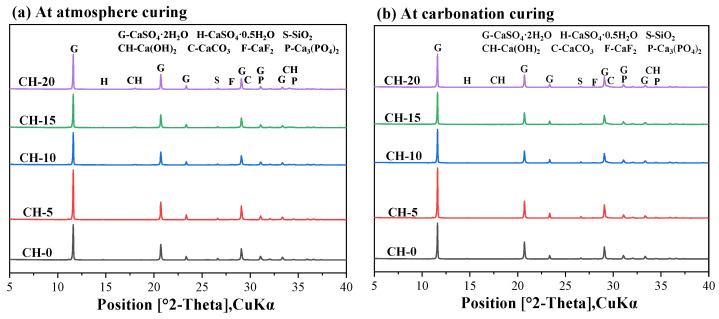
The XRD patterns of pastes: (**a**) atmosphere-cured specimens, (**b**) carbonation-cured specimens.

Figure 9 displays the XRD patterns of the pastes exposed to atmosphere curing and carbonation curing for 14 days.

In all the samples, we can observe the presence of CaSO_4_·2H_2_O, CaCO_3_, CaF_2_, Ca_3_(PO_4_)_2_ and impurity SiO_2_. It is hard to detect CaSO_4_·0.5H_2_O, which here hydrated to form CaSO_4_·2H_2_O. The characteristic peaks of Ca_3_(PO_4_)_2_ and CaF_2_ were detected, suggesting that CH could solidify harmful impurities into insoluble substances in HPG [18]. In Figure 9a, diffraction peaks of CH were apparent in the atmosphere-cured pastes, and CaCO_3_ was also identified due to natural carbonation. In Figure 9b, CaCO_3_ was detected in the carbonation pastes and Ca(OH)_2_ could hardly be detected, demonstrating that the Ca(OH)_2_ was almost carbonated to form CaCO_3_. The gypsum peak overlaps with the CaCO_3_ peak at around 29°, and TG-DTG analysis could help to further analyze the change in hydration products.

#### 3.4.2. TG-DTG Analysis

**Figure 10:**
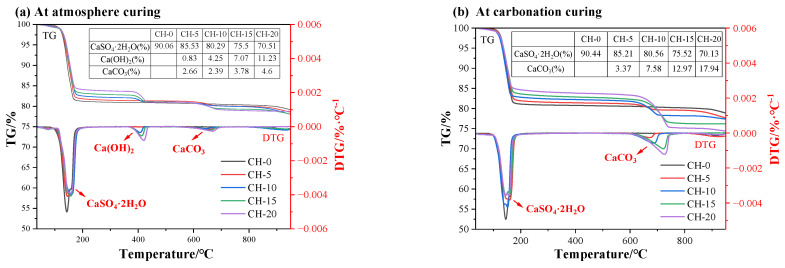
The TG/DTG curves of pastes: (**a**) atmosphere-cured specimens; (**b**) carbonation-cured specimens.

The DTG/TG analysis of HPG paste powder samples after 14 d of atmosphere curing and carbonation curing was used to calculate the amounts of hydration products. For samples under atmosphere curing, as shown in Figure 10a, three clear stages of mass loss occur at 100–190 °C, 360–450 °C, and 540–700 °C due to the decomposition of CaSO_4_·2H_2_O [35], Ca(OH)_2_ and CaCO_3_ [36] (Ca(OH)_2_ was lightly carbonated in air), respectively.

As displayed in Figure 10b, the carbonated specimens have weak dehydration peaks of CH, suggesting that most of the CH was consumed. CaCO_3_ decarbonation at higher temperatures from 670 °C to 730 °C implies its good crystallization [37]. Furthermore, a higher CH content resulted in a higher decarbonation peak, also revealing the formation of well-crystallized CaCO_3_ [38]. 

### 3.5. Microstructure Analysis

**Figure 11:**
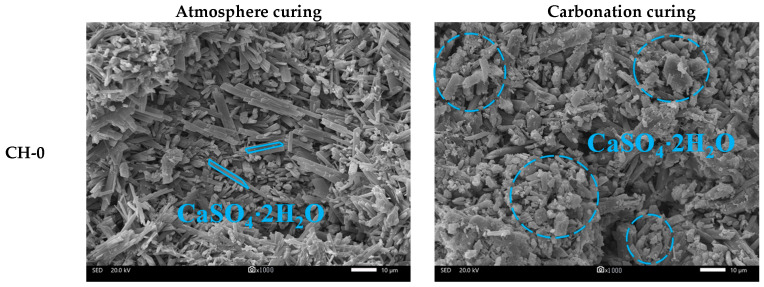

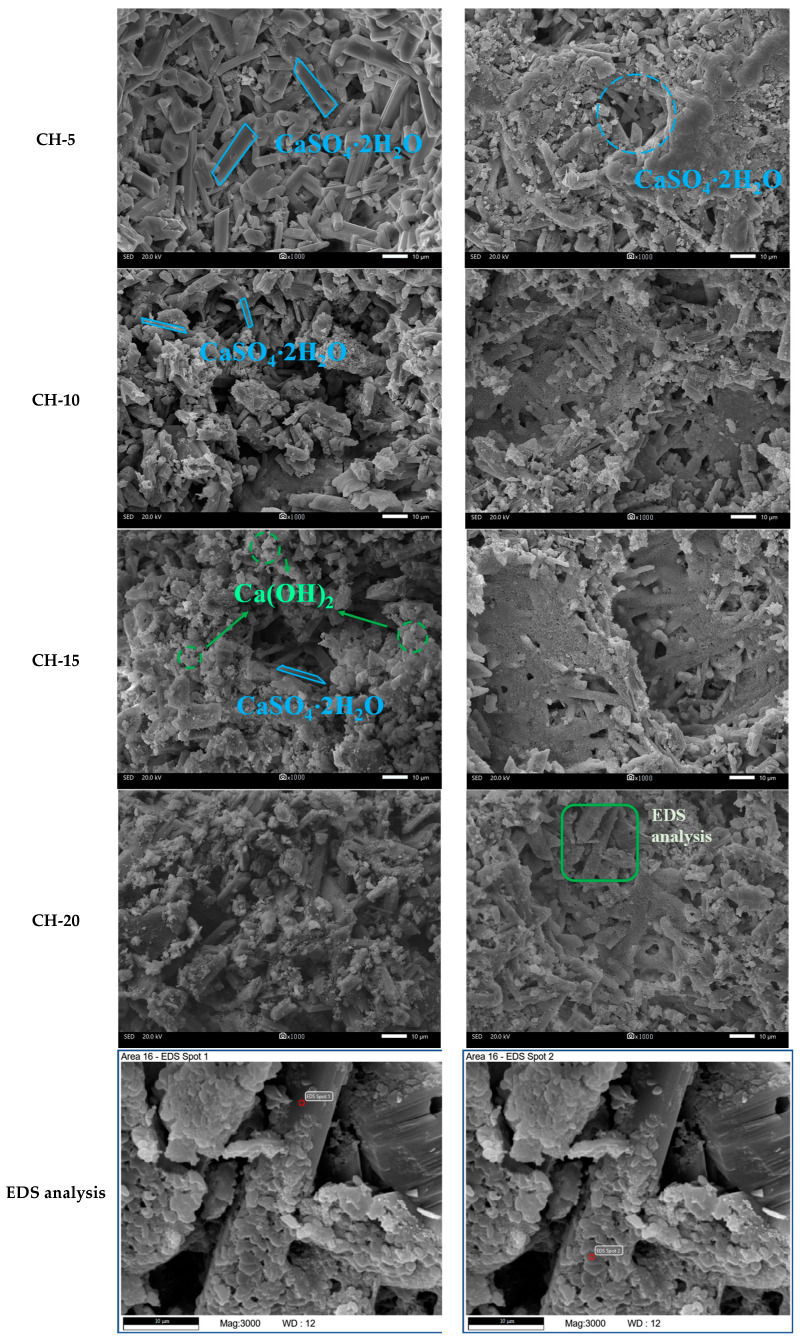

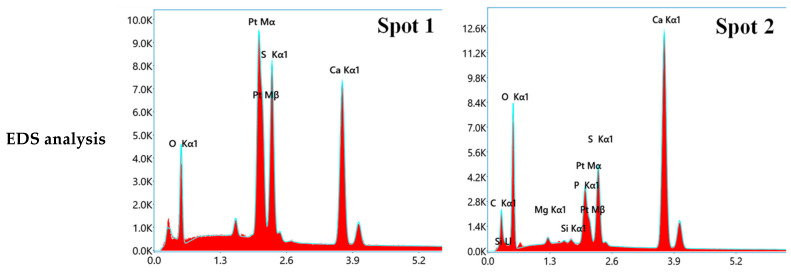
The SEM-EDS analysis of HPG pastes.

Figure 11 displays the SEM-EDS analysis of HPG pastes after 14 d of atmosphere curing and carbonation curing.

In the atmosphere-cured group, it was evident that the appearance of gypsum crystals was altered with CH addition from interlaced needle-like (CH-0) to short prism-like (CH-5), and the length–radius ratio decreased [39]. With increasing dosages of CH, the gypsum crystal shapes recovered to some extent (CH-10, CH-15), while the number of gypsum crystals decreased. Fewer gypsum crystals formed effective contact with each other, and a large amount of CH remained uncarbonated, resulting in a loose microstructure.

As for carbonation-cured samples, firstly, the gypsum crystals in the pure HPG paste showed poor crystallization (CH-0) and the addition of CH restored the crystal growth of gypsum (CH-5). Secondly, it can be seen that lots of nanoscale CaCO_3_ crystals like fish’s scales covered the gypsum crystals to form a dense carbonate layer (shown in EDS analysis). Thirdly, CaCO_3_ crystals filled in the pores to make the microstructure denser. However, the insufficient amount of CH (CH-5) limited the coverage of CaCO_3_. With increasing dosages of CH (CH-10, CH-15 and CH-20), the carbonation-cured samples showed a dense microstructure.

### 3.6. Pore Structure

**Figure 12:**
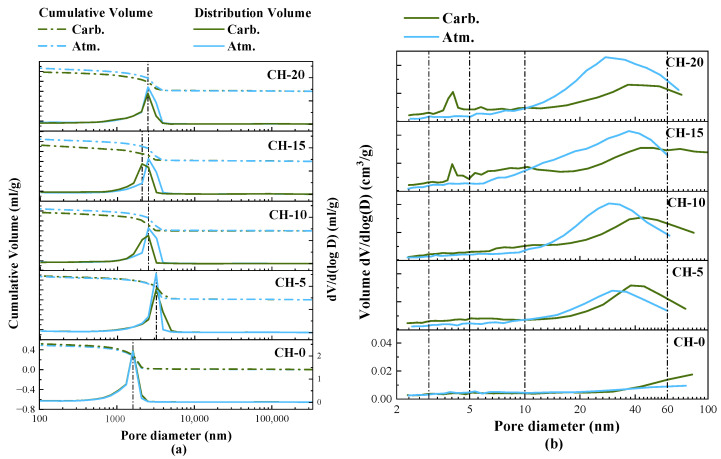
Pore size distributions of HPG pastes: (**a**) pore (>100 nm) distributions analyzed by MIP; (**b**) pore (<100 nm) distributions analyzed by N_2_ physisorption.

The pore structure of HPG-hardened pastes can be used to interpret the macroscopic performance (mechanical strength [40] and water resistance [41]). In accordance with the International Union of Pure and Applied Chemistry (IUPAC) pore classification [42] for <2 nm micropores, 2–50 nm mesopores, and >50 nm macropores, mercury intrusion porosimetry (MIP) was used to analyze pores >100 nm and N_2_ physisorption measurement was used to analyze pores <100 nm in the study [43]. The distributions of pores are presented in Figure 12.

According to the results obtained from MIP in Figure 12a, under atmosphere curing, fewer changes were seen in the cumulative porosity, and the most probable aperture (MPA) shifted to a larger size with the addition of CH. Under carbonation curing, the cumulative porosity of pure HPG paste was slightly increased, and the cumulative porosity of HPG + CH pastes was significantly decreased compared to atmosphere curing. Compared to the strength, the changes in accumulated porosity showed a higher correlation with the softening coefficient of HPG pastes. Carbonation-cured CH-15 achieved the highest softening coefficient with the minimum cumulative porosity. The number of MPA of carbonated HPG + CH pastes was reduced compared to atmosphere curing, and the size of MPA of carbonated CH-15 was smaller than that of atmosphere curing. In summary, carbonated HPG + CH pastes reduced the number of pores and refined the distribution of pores compared to atmosphere curing.

The pore distributions between 2 and 100 nm obtained by N_2_ physisorption analysis using a BJH model are displayed in Figure 12b. The atmosphere-cured pastes exhibited higher pore volumes with increasing CH content. For the carbonation-cured pastes, the volumes of pores between 10 and 60 nm remarkably decreased when CH exceeded 5 wt.%, and a second peak appeared between 3 and 5 nm in both CH-15 and CH-20. This suggests that sufficient CH replacement could reduce the mesopore size of HPG pastes and expand the pore distribution range under carbonation curing. This is related to the agglomeration and filling effects of CaCO_3_, associated with the SEM results.

## 4. Discussion

**Figure 13:**
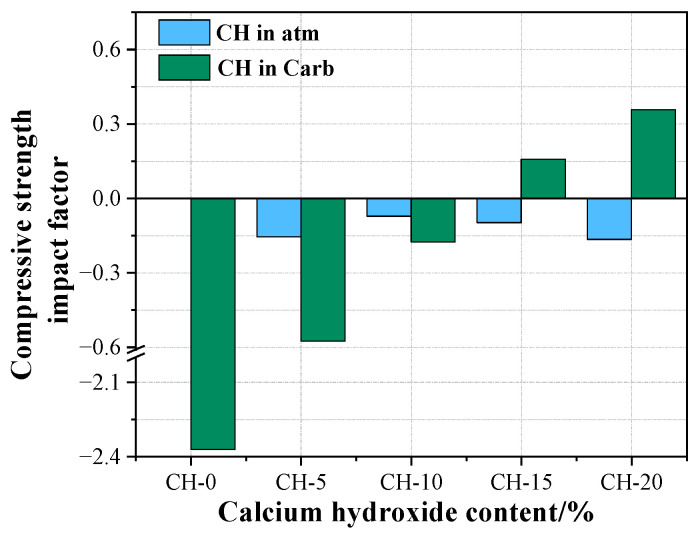
Compressive strength impact factor.

To visually analyze the effects of adding CH and carbonation on the properties of HPG pastes, we used Equation (3) to calculate their compressive strength impact factor, and displayed the results in Figure 13. The positive and negative values of compressive strength influencing factors represent the positive and negative influence on compressive strength of the reference group.

(3) $$\lambda =\frac{S_{t}}{S_{\mathrm{CH}\text{-}0}}$$

where *λ* is the compressive strength impact factor; *S_t_* (MPa) is the compressive strength of samples; *S*_CH-0_ (MPa) is the compressive strength of CH-0 under atmosphere curing.

### 4.1. The Effects of CH on the Performance of HPG

The effects of CH on the performance of HPG are analyzed according to the atmosphere-cured pastes. As revealed by XRD and SEM analysis, incorporating CH into HPG could generate Ca_3_(PO_4_)_2_ and CaF_2_, which coated the surfaces of HPG and gypsum nuclei, impeding the dissolution of HPG, and concurrently slowing down the formation of gypsum nuclei and suppressing the growth of gypsum crystals [44]. Therefore, adding CH into HPG sharply prolonged the setting time, and presented a negative influence on compressive strength and water resistance (showed in Figure 7 and Figure 13). With the increase in CH, P and F were completely neutralized, and excess CH particles preferentially adsorbed Ca_3_(PO_4_)_2_ and CaF_2_, which mitigated their negative impacts on the growth of gypsum crystals [19]. Furthermore, the residual CH particles could act as nuclei for the crystallization of gypsum through the seeding effect [20]. This is why the setting time gradually decreased when the CH content exceeded 5 wt.%. However, during this process, there was a dilution effect that decreased the number of gypsum crystals, and a large amount of CH remained uncarbonated, which jointly led to a larger pore size and a loose microstructure, and reduced the performance of HPG (showed in Figure 13). However, the structure is conducive to the carbonation of CH.

### 4.2. The Effects of Carbonation Curing on the Performance of HPG + CH Pastes

In Figure 11, Figure 12 and Figure 13, it can be observed that carbonation curing has a heavy negative influence on the strength of the pure HPG, with poor crystallization and a higher cumulative porosity. This is because carbonation curing has an adverse effect on the gypsum crystallization, similar to the effect of phosphoric acid on HPG [39]. The samples were carbonated at all levels of CH used as an HPG replacement. Since the coverage of CaCO_3_ crystals formed from the residual CH after neutralization was limited, 5 wt.% CH addition showed a negative value of compressive strength influencing factor, as shown in Figure 13. With the increase in CH addition under carbonation curing, a greater number of insoluble CaCO_3_ crystals were precipitated (about 3.37~17.94% from TG analysis) with an 11.4% increase in solid volume [45]. CaCO_3_ aggregation not only formed a compact layer of carbonate salts on the surface of gypsum crystals, but also filled in the pores and around the pore surfaces of the gypsum crystals (shown in SEM analysis), which led to a smaller pore size, a lower porosity and a dense microstructure. This is the reason why the compressive strength influencing factor value of HPG + CH pastes under carbonation curing changed from negative to positive and increased with the increase in CH in Figure 13. This is also of great significance for enhancing the water resistance of gypsum. The dense carbonate layer on the surface of gypsum crystals could prevent the dissolution of gypsum, and the aggregation of CaCO_3_ filling in the pores and around the pore surfaces could block both the entry and exit of moisture. As a result, the collaborative effects of carbonation curing and CH addition cancel out the harm that a single factor causes.

**Figure 14:**
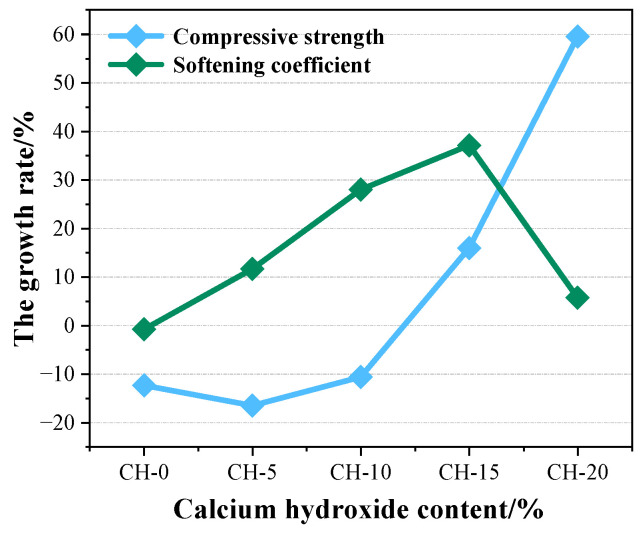
The growth rate of compressive strength and softening coefficient of carbonation-cured specimens compared to atmosphere-cured specimens.

To select the optimal dosage of CH, Figure 14 displays the growth rates of the compressive strength and softening coefficient of carbonation-cured specimens compared to atmosphere-cured specimens.

As revealed by Figure 14, the growth rate of the compressive strength initially exhibited a negative value, followed by an increase to a positive value with the increment of CH content. Concurrently, the growth rate of the softening coefficient first improved and then declined when the CH content exceeded 15 wt.%. The negative values of the growth rate of compressive strength in CH-0, CH-5 and CH-10 are due to the adverse effects of carbonation curing and the presence of fewer CaCO_3_ crystals. The lower softening coefficient of CH-20 is expected to be attributed to the greater CH addition. A large amount of CaCO_3_ crystals was not enough to make up for the structural damage caused by the reduction in gypsum crystals resulting in pore coarsening. In Figure 14, the carbonation-cured CH-15 showed optimal comprehensive performance with a softening coefficient of 0.65 increased by 37.1%, and a compressive strength of 17.16 MPa increased by 16.2%. The modified HPG is suitable for making blocks [46], tiles, plastering and masonry work, making glass/sisal reinforced boards and so on [47]. The synergistic effects including the neutralizing, seeding and diluting effects related to CH and the filling effects related to CaCO_3_ may be optimized. CH-15 achieved the densest microstructure and the minimum cumulative porosity.

There are some studies on improving the water resistance of HPG. Fornés et al. [48] modified HPG with 0.3% metallurgical sludge, and the softening coefficient was 0.53. According to Jin et al. [49], the compressive strength and softening coefficient of HPG can be improved to 21.5 MPa and 0.66 with the content of 20% sulphoaluminate cement. The results show that adding excessive CH into HPG and combining with carbonation curing is an effective modification process with low costs, significant enhancement effects and low carbon emissions. The modified effects could be further enhanced by dispersing CH more uniformly, adding a crystal regulator of CaCO_3_ and adding other carbonatable alkaline substances.

### 4.3. Carbon Footprint and Energy Consumption of Carbonated HPG + CH Pastes


(4) $$2CaSO_{4}\cdot 0.5H_{2}O+{Ca\left(OH\right)}_{2}+CO_{2}+{2H}_{2}O\to 2CaSO_{4}\cdot 2H_{2}O+CaCO_{3}$$

The carbonation process can be expressed by Equation (4). For the comprehensive assessment of the carbon footprint and energy consumption of HPG pastes, the normalized saturated conditions’ compressive strength carbon footprint Cs can be calculated by Equation (5), and the normalized saturated conditions’ compressive strength energy consumption (heat and electricity) Es can be calculated by Equation (6).

(5) $$C_{s}=\frac{\sum_{i=1}^{n}N_{i}\cdot m_{i}-C_{C}}{R_{C}}$$

(6) $$E_{s}=\frac{\sum_{i=1}^{n}W_{i}\cdot m_{i}}{R_{C}}$$

where $N_{i}$ (kgCO_2_/kg) and $W_{i}$ (MJ/kg) are the carbon emission and the energy consumption of the *i*-th raw material, as shown in Table 5; $m_{i}$ (kg·m^−3^) is the mass of raw material for HPG pastes based on unit volume; $C_{C}$ (kgCO_2_·m^−3^) is the carbon capture of HPG pastes based on unit volume calculated from TG analysis; $R_{C}$ (MPa) is the saturated conditions’ compressive strength.

The carbon footprint and energy consumption of carbonated HPG + CH pastes are shown in Table 6. CH-15 exhibited the lowest normalized saturated conditions compressive strength carbon footprint and smallest normalized saturated conditions compressive strength energy consumption at 110.76 kgCO_2_·m^−3^·MPa^−1^ and 1083.81 MJ·m^−3^·MPa^−1^, respectively. These findings highlight that the carbonation of the HPG pastes with 15 wt.% CH is an environmentally friendly option.

Furthermore, CH can be replaced with solid wastes, with CH as the main component (such as calcium carbide slag generated in the production of acetylene). Phosphoric acid plants can use an HPG and calcium carbide slag composite system combined with exhaust gas to prepare gypsum products, further reducing energy consumption and CO_2_ emissions, to achieve the treatment of waste and to yield cleaner gypsum products.

## 5. Conclusions

This study investigates the collaborative effects of calcium hydroxide (CH) addition and carbonation curing on the performance of hemihydrate phosphogypsum (HPG) pastes. The conclusions could be summarized as follows:

1. The addition of 5 wt.% to 20 wt.% CH could prolong the setting times and decrease the fluidities of the fresh HPG pastes, and the compressive strength and the softening coefficient of hardened HPG pastes were decreased by about 9–37% and 13%, respectively;
2. CaF_2_ and Ca_3_(PO_4_)_2_ could be identified in the HPG + CH pastes due to neutralization;
3. Carbonation curing could significantly recover the compressive strength and water resistance of the hardened HPG + CH pastes. The carbonated HPG + 15 wt.% CH paste demonstrated the best performance—compared with the reference paste under atmosphere, the compressive strength and softening coefficient increased by 16.2% and 37.1%, respectively;
4. A large number of nanoscale CaCO_3_ crystals were identified to fill the pores of the carbonated HPG + CH pastes and cover the surface of CaSO_4_·2H_2_O crystals. This refinement and coverage caused the improvement of compressive strength and water resistance;
5. The carbonated HPG + 15 wt.% CH paste is also an environmentally friendly option, exhibiting the lowest normalized saturated conditions compressive strength carbon footprint and energy consumption, with 13.3% and 6.2% reductions compared to pure HPG paste.

## Figures and Tables

**Table 1:** The physical and mechanical properties of HPG.

	Radionuclide	Initial Setting Time (min)	Final Setting Time (min)	2 h Flexural Strength (MPa)	2 h Compressive Strength (MPa)
Test results	I_Ra_ = 0.3, I_c_ = 0.3	7	16	3.3	8.7

**Table 2:** Chemical composition of raw material (wt.%).

Composition	SO_3_	CaO	SiO_2_	F	P_2_O_5_	Al_2_O_3_	Fe_2_O_3_	Na_2_O	TiO_2_	K_2_O	SrO	BaO
HPG	46.69	35.96	5.598	1.8	1.03	0.755	0.36	0.085	0.0742	0.0729	0.0728	0.071
CH	0.292	82.56	0.98	-	0.01	0.408	0.239	-	0.022	0.0807	0.0211	-

**Table 3:** Mix proportion of paste mixtures (wt.%).

Mixtures Notation	HPG	Ca (OH)_2_	Retarder XK (By Mass of HPG)	Water to Powder Ratio
CH-0	100	0	0.01	0.58
CH-5	95	5		
CH-10	90	10		
CH-15	85	15		
CH-20	80	20		

**Table 4:** PDF code and ICSD collection code used for qualitative analysis of the diffraction patterns.

Compound Name	Chemical Formula	PDF Code	ICSD Collection Code	Author [Ref.]
Gypsum	CaSO_4_·2H_2_O	00-033-0311	92567	Schofield [28]
Quartz	SiO_2_		156196	Ikuta [29]
Portlandite	Ca(OH)_2_	00-004-0733	73467	Desgranges [30]
Calcite	CaCO_3_	00-005-0586	80869	Goergens [31]
Calcium Phosphate	Ca_3_(PO_4_)_2_		10941	Hanawalt [32]
Calcium Fluoride	CaF_2_	01-077-2096	60371	Batchelder [33]

**Table 5:** Carbon footprint factors.

Raw Materials		CO_2_ Emission (kgCO_2_/kg)	[Ref.]	Energy Consumption (MJ/kg)	[Ref.]
HPG	Formation of PG	0.18	[50]	1.0	[51]
	Calcination of PG	0.13	[52]	1.8	[52]
CH		0.78	[52]	5.3	[52]

**Table 6:** Carbon footprint and energy consumption of carbonated HPG + CH pastes based on unit volume.

HPG Pastes	CO_2_ Emission (kgCO_2_·m^−3^)		CO_2_ Capture $C_{C}$ (kgCO_2_·m^−3^)	$C_{s}$ (kgCO_2_·m ^−3^·MPa^−1^)	Energy Consumption (MJ·m^−3^)		$E_{s}$ (MJ·m^−3^ ·MPa^−1^)
	$N_{HPG}\cdot m_{HPG}$	$N_{CH}\cdot m_{CH}$			$W_{HPG}\cdot m_{HPG}$	$W_{CH}\cdot m_{CH}$	
CH-0	1174.61	0.00	0.00	127.68	10,609.38	0.00	1153.19
CH-5	1115.88	147.77	54.89	159.05	10,078.91	1004.10	1458.29
CH-10	1057.15	295.55	124.41	136.48	9548.44	2008.20	1284.07
CH-15	998.42	443.32	213.34	110.76	9017.97	3012.30	1083.81
CH-20	939.69	591.09	296.47	128.57	8487.50	4016.41	1302.49

## Data Availability

The data presented in this study are available on request from the corresponding author.
